# Cultivated Oral Mucosal Epithelial Transplantation for Limbal Stem Cell Deficiency: A Scoping Review of Indications, Platforms, Outcomes and Safety

**DOI:** 10.3390/jcm15031134

**Published:** 2026-02-01

**Authors:** Konstantinos Papadopoulos, Mohamed Elalfy, Hasan Naveed, Sokratis Zormpas, Artemis Matsou

**Affiliations:** 1 Guy’s and St Thomas’ NHS Foundation Trust, London SE1 7EH, UK; konstantinos.papadopoulos@nhs.net; 2 Corneoplastic Unit, Queen Victoria Hospital, East Grinstead RH19 3DZ, UK; m.elalfy@nhs.net (M.E.); hasan.naveed@nhs.net (H.N.); 3 The Research Institute of Ophthalmology, Cairo 3725102, Egypt; 4 King’s College Hospital, London SE5 9RS, UK; sokratis-zor@hotmail.com

**Keywords:** limbal stem cell deficiency, COMET, cultivated oral mucosal epithelial transplantation, CLET, SLET, ocular surface reconstruction, amniotic membrane

## Abstract

**Background:** Cultivated oral mucosal epithelial transplantation (COMET/CAOMECS) is an autologous, immunosuppression-sparing option for ocular surface reconstruction in limbal stem cell deficiency (LSCD). After two decades, indications, platforms and outcome definitions vary, and COMET’s position relative to limbal-derived epithelium remains uncertain. **Methods:** We conducted a PRISMA-ScR scoping review of human clinical studies (PubMed, 2000–30 December 2025) with hand-searching and regulatory sources. Eligible reports included COMET/CAOMECS series and comparative cohorts (CLET/ACLET, SLET, KLAL/CLAL). The primary outcome was anatomical success (stable epithelialised cornea without recurrent persistent epithelial defect, progressive conjunctivalisation or uncontrolled neovascularisation at last assessment). Given heterogeneity in definitions and analytic frames (fixed-time vs. Kaplan–Meier [KM]), results were synthesised narratively by indication and platform. **Results:** Twenty-five reports (893 eyes; 821 patients) were included. Aetiologies were predominantly burns and SJS/TEN. Across amniotic membrane-based mixed-aetiology series, 12-month anatomical success clustered around 55–70%. Aggregated descriptively across COMET eyes, 211/467 (45%) had a stable surface at last follow-up. Epithelialisation was generally rapid in quiet AM-based reconstructions and slower with severe adnexal disease or carrier-free platforms. Mean BCVA improved from 1.8 ± 0.7 to 1.4 ± 0.7 logMAR (471 eyes); ≥2-line gains occurred in 308/471 (65.4%). A matched comparison suggested better 12-month survival, less neovascularisation and better BCVA with substrate-free versus AM-carried COMET; a biomaterial-/feeder-free platform reconstructed most eyes but failed more often with four-quadrant symblepharon. Observational comparative cohorts suggested higher surface survival and average visual gain with limbal-derived epithelium, at the cost of systemic immunosuppression. **Conclusions:** In appropriately selected bilateral LSCD, COMET offers immunosuppression-sparing reconstruction with moderate, durable surface stability and clinically meaningful visual gains when performed on a quiet, optimised surface. Platform refinements—particularly substrate-free constructs—and prospective, indication-defined comparative studies with harmonised outcomes are needed to define COMET’s role relative to limbal-derived epithelium.

## 1. Introduction

Limbal stem cell deficiency (LSCD) is a vision-threatening ocular surface disease defined by loss or dysfunction of limbal epithelial stem/progenitor cells, with consequent failure of corneal epithelial homeostasis leading to conjunctivalisation, recurrent epithelial breakdown, stromal neovascularisation, scarring and sight-threatening surface instability [1,2]. The 2019 Global Consensus standardised definition, classification and staging, facilitating more rigorous diagnosis, reporting and trial design [3]. Its aetiologies span alkali and thermal injuries, Stevens–Johnson syndrome and toxic epidermal necrolysis (SJS/TEN), ocular cicatricial pemphigoid (OCP), aniridia, severe contact lens toxicity and iatrogenic causes. Surgical restoration of a self-renewing corneal epithelium is the therapeutic cornerstone in moderate–severe disease [1,2,3,4].

Over three decades, surgical management of LSCD has evolved from conjunctival–limbal autograft (CLAU) to ex vivo cultivated limbal epithelial transplantation (CLET) and simple limbal epithelial transplantation (SLET). Autologous CLET achieved first-in-class regulatory approval in the EU as Holoclar^®^ in 2015, establishing a benchmark for cell-based corneal surface restoration [5]. SLET, introduced in 2012, combines the biological advantages of limbal explant outgrowth with minimal donor harvest and has become the preferred option for many unilateral cases [6].

For bilateral LSCD, autologous limbal tissue is unavailable. Allogeneic strategies (living-related CLAL or KLAL) demand long-term systemic immunosuppression, which can have associated risks [7]. Cultivated oral mucosal epithelial transplantation (COMET), which is the expansion of a patient’s oral epithelial cells into a transplantable sheet, was conceived to provide an autologous, immunosuppression-sparing solution [8,9,10]. Since the original clinical series in the early 2000s, COMET has matured into an approved product class in Japan (Ocural^®^ 2021; Sakracy^®^ 2022), reflecting real-world translatability of the platform [11,12]. In parallel, laboratories have refined xenobiotic-free culture methods and feeder-free approaches and emerging techniques such as SOMET (simple oral mucosal epithelial transplantation) are being explored [13].

However, the key question of which patients benefit most from COMET remains, as well as how its anatomical and functional outcomes compare with autologous CLET (or SLET in unilateral disease) and with allogeneic options.

Given the breadth of indications, surgical platforms, outcome definitions, and follow-up durations reported across the COMET literature, we chose to perform a scoping review rather than a systematic review or meta-analysis. Scoping reviews are particularly suited to mapping heterogeneous evidence bases, clarifying how interventions are applied in practice, and identifying knowledge gaps, rather than estimating pooled effect sizes or comparative efficacy. In the context of COMET, where clinical heterogeneity and methodological variability are considerable, this approach allows a structured synthesis of indications, platforms, outcomes, and safety while avoiding overinterpretation of non-comparable data.

## 2. Methods

We conducted a scoping review to map indications, surgical platforms, outcomes, and safety of cultivated oral mucosal epithelial transplantation (COMET/CAOMECS) for the surgical rehabilitation of limbal stem cell deficiency. The review followed PRISMA-ScR guidance for planning and reporting. No statistical meta-analysis or formal risk-of-bias assessment was planned, consistent with scoping methodology. This reflects the predominance of non-randomised case series and observational cohorts in the COMET literature. As such, the findings of this review should be interpreted as descriptive and hypothesis-generating. Comparative observations and platform-related signals are not intended to establish causality or procedural superiority.

We included human clinical reports (any design) in which COMET (or equivalent autologous oral mucosal epithelial cell sheets) was used to treat LSCD, with clinical outcomes reported. Randomised or non-randomised trials, prospective or retrospective cohorts, and case series that reported clinical outcomes of COMET/CAOMECS were eligible, as were comparative cohorts against CLET/ACLET, SLET, Keratolimbal allograft (KLAL)/conjunctival limbal allograft (CLAL), or amniotic membrane alone. We excluded animal studies, laboratory studies without human clinical outcomes, technique notes without outcomes and single-eye case reports. Studies focused on non-cultivated oral mucosal transplantation (OMET/SOMET) were considered for context only and not pooled with COMET outcomes.

Data sources included MEDLINE via PubMed from 1 January 2000 to 30 December 2025, complemented by hand-searching reference lists of included papers and key reviews and regulatory sources for context (PMDA/EMA communications). The core PubMed strategy combined controlled vocabulary and text words for LSCD and COMET/CAOMECS and their variants (example (“limbal stem cell deficiency” [MeSH] OR “limbal stem cell*” or LSCD) AND (COMET or CAOMECS or “cultivat* oral mucos*” OR “oral mucosal epithelial sheet*” OR “oral mucosal epithelial transplant*”)), limiting the scope to humans and the English language. Notably, we acknowledge that the omission of additional databases such as Embase, Scopus, or Web of Science may have resulted in incomplete coverage, particularly of translational studies.

Two reviewers (K.P., A.M.) independently screened titles/abstracts and then full texts against the eligibility criteria. Disagreements were resolved by a third reviewer (S.Z.). Duplicates were removed before screening. A standardised extraction form captured study design, sample size (eyes/patients), LSCD aetiology and extent, graft manufacturing platform (AM-supported, substrate-free/fibrin dish, temperature-responsive/ carrier-free, biomaterial-/feeder-free), surgical adjuncts (AM, mitomycin-C, lid/adnexal procedures), postoperative therapy (topical/systemic steroids, immunosuppression), follow-up duration, definitions of anatomical success, visual outcomes, time to epithelial closure, need for re-intervention and adverse events.

To align with commonly used definitions in the COMET literature, the primary outcome was anatomical success, defined as a stable, epithelialised corneal surface without recurrent persistent epithelial defect (PED) for ≥3 months and without progressive conjunctivalisation or uncontrolled neovascularisation at the last follow-up. However, we acknowledge that this timeframe does not equate to long-term disease control in a chronic condition such as LSCD. As such, this definition should be viewed as an early anatomical endpoint rather than a surrogate for durable long-term survival.

The secondary outcomes were change in best-corrected visual acuity (BCVA, logMAR), time to epithelial closure, recurrence of LSCD and safety endpoints (e.g., infectious keratitis, graft melt/failure, glaucoma, cataract progression, symblepharon recurrence, donor-site oral complications, dysplasia on histology). Given clinical and methodological heterogeneity in indications, platforms, outcome definitions, and analytic frames (fixed-time proportions vs. Kaplan–Meier survival), we performed a structured narrative synthesis and evidence map with subgroups by indication (burns, SJS/TEN, OCP, aniridia, acute PED, fornix reconstruction) and by platform. The search was last updated on 30 December 2025. Ethics approval was not required for this review.

## 3. Results

Twenty-five reports [2,8,9,10,14,15,16,17,18,19,20,21,22,23,24,25,26,27,28,29,30,31,32,33,34] met the eligibility criteria. Table 1 shows the studies’ characteristics. Thirteen studies [8,17,21,23,24,26,27,29,30,31,32,33,34] were prospective in design, ten [2,9,10,14,16,18,20,22,25,28] retrospective, one histopathological [19] and one combined translational/prospective [15]. Seventeen were clinical series of cultivated oral mucosal epithelial transplantation (COMET/CAOMECS) reporting outcomes and three were comparative cohorts that included a COMET arm. Studies originated predominantly from Japan, France, Thailand, India, Korea, the UK and Singapore. Some studies, particularly from Japanese centres, represent longitudinal or subgroup analyses of overlapping cohorts and were interpreted narratively to avoid double-counting. In total, 893 eyes of 821 patients were included (64.7% male, 35.3% female). The mean age of the cohort was 45.8 ± 13.9 years (range 2.5 to 86), 44.8 ± 12.9 years for men and 47.4 ± 15.4 years for women.

### 3.1. Aetiology

Table 2 lists the aetiology of LSCD within the respective studies. The most common aetiology was corneal burn (chemical, thermal or unspecified: 346/893 eyes, 38.8%), followed by Stevens–Johnson syndrome/ TEN (SJS/TEN: 250/893, 28.0%). Ocular cicatricial pemphigoid (OCP: 72/893, 8.1%) and aniridia (71/893, 8.0%) were the next most frequent. The remaining 17.1% were classified as miscellaneous (trachoma, post-keratitis, idiopathic, Lyell syndrome, rosacea keratitis, congenital aniridia, contact lens hypoxia, neuroparalytic keratitis, graft-versus-host disease, squamous cell carcinoma, gelatinous drop-like dystrophy, multiple ocular surgeries, advanced pterygium, ocular trauma, cystinosis, hepatitis, radiation keratopathy, Salzmann’s degeneration, and drug toxicity).

### 3.2. Preoperative Interventions

Across fourteen studies [8,10,13,14,18,19,20,21,22,23,24,25] that reported prior surgery, 123/256 eyes (48%) had ≥1 previous interventions. Among eyes with the type of prior intervention recorded, there were 150 interventions. Amniotic membrane transplantation (AMT) accounted for 51/150 (34%), corneal graft for 16/150 (10.7%), and 83/150 (55.3%) were categorised as “other” (e.g., tenonplasty, fibrin glue, symblepharon release).

### 3.3. Surgical Approaches and Platforms

Early programmes relied on amniotic membrane (AM)-supported sheets whereas later series introduced substrate-free (fibrin-dish), temperature-responsive/carrier-free, and biomaterial-/feeder-free sheets. Across all included surgical series, COMET was used in 467 eyes (52%), CLET in 210 (24%), and SLET in 47 (5.3%), whilst 10 eyes underwent (buccal mucosal epithelial cell transplantation (BMECT) (Priya et al.) and 26 underwent CAOMECS (Burillon et al.). Three studies (two by Sotozono et al. and one by Shimazaki et al.) reported simultaneous COMET with AM transplantation (125 eyes, 14%).

### 3.4. Postoperative Regimen and Follow-Up

Postoperative care was broadly similar across series (topical antibiotic and corticosteroid with taper). Systemic immunosuppression was not routine for COMET (reported in one study by Shimazaki), though short systemic steroid courses were used in eleven studies [8,10,17,19,20,21,26,27,28,29,32]. One study [18] included pre- and postoperative intravenous steroids. The mean follow-up was 20.8 ± 15.6 months (reported for 521 eyes; range 0.3–85.6 months).

### 3.5. Anatomical Outcomes

A stable corneal surface was reported in 211/467 (45%) COMET eyes at the last follow-up. In the AM-based mixed-aetiology series, 12-month anatomical success typically fell in the mid-range (~55–70%) under each study’s definition (stable epithelialisation without progressive conjunctivalisation or uncontrolled neovascularisation). In the first human series, Nakamura et al. (2004)achieved complete epithelial cover in all six eyes within 48 h, with stable surfaces thereafter and only mild peripheral neovascularisation while VA improved in all eyes [8]. Inatomi et al. (2006) reported stable, transparent, epithelialised corneas in 10/15 (67%), with 14/15 re-epithelialised by day two [34]. Satake et al. (2011) reported Kaplan–Meier “stable surface” survival of 64.8% at 1 year, 59.0% at 2 years, and 53.1% at 3 years [9]. Early failure was driven by PED and fibrovascular invasion within three months. The multicentre CAOMECS study (Burillon et al. 2012) met its day-360 primary endpoint in 16/25 eyes (one lost to follow-up) with improvement in symptoms [32]. In Thailand, Prabhasawat et al. (2016) reported 15/20 (75%) “clinical success” with KM estimates of 79.3% at 1 year and 70.5% at 4 years; 19/20 eyes closed in the first postoperative week, and one perforation occurred due to eyelid abnormality [23]. Indication-specific cohorts were comparable: Sotozono et al. (2013) followed 46 eyes for 48 ± 43 months, reporting KM stability of ≈74% at 1 year declining to ≈39% at 7 years with central corneal clarity tracking the same trajectory. Dobrowolski et al. (2015) [22] (aniridia, 17 eyes) achieved a “regular transparent epithelium” in 76.4% with 88.2% VA improvement [28]. Sotozono et al. (2014) (acute PED, 10 eyes) achieved closure in all treated eyes with sustained stability [14].

### 3.6. Platform Effects

Technique appears to influence outcome. In a matched Kyoto case–control comparison, substrate-free (fibrin-dish) sheets achieved 12-month success of 62.5% versus 43.8% for AM-based COMET, with significantly better survival (KM, *p* = 0.046), less neovascularisation and better BCVA at all time-points. A biomaterial-/carrier-free, feeder-free clinical trial (Kim et al., 2018 [29]; 8 eyes) reconstructed 6/8 totally deficient eyes with a mean time to epithelialisation of 53.6 days, and 62.5% of eyes gaining at least two lines of visual acuity. Failures clustered in eyes with four-quadrant symblepharon. These findings suggest that, where feasible, non-AM platforms can reduce early fibrovascular encroachment and yield stronger early functional gains; however, available evidence remains limited and exploratory. Platform-related comparisons are derived from small, predominantly single-centre cohorts. High-quality prospective head-to-head comparisons are needed.

### 3.7. Time to Epithelialisation

Epithelialisation kinetics diverged by platform and ocular surface state. In quiet AM-based reconstructions, re-epithelialisation was rapid: 48 h in Nakamura et al. [8], postoperative day 2 in 14/15 eyes in Inatomi et al. [34], and within the first week in 19/20 eyes in Prabhasawat et al. [23]. In contrast, complex cicatrising surfaces and carrier-free constructs required weeks (Kim et al.: mean 53.6 days). In the acute PED setting (Sotozono et al.) prompt and durable closure in all eyes was achieved [14].

### 3.8. Visual Outcomes

Vision improved in most series but was frequently limited by stromal opacity and neovascularisation in severe cicatrising disease. Across studies reporting VA, pre- and postoperative VA were 1.8 ± 0.7 and 1.4 ± 0.7 logMAR, respectively (471 eyes). An improvement of ≥2 lines was observed in 308/471 eyes (65.4%). The absent or incomplete description of VA measurement methodology in several reports limited direct comparison across studies.

### 3.9. Comparative Cohorts (Positioning COMET)

Table 3 describes the comparative studies. Real-world comparisons consistently favoured limbal-derived epithelium for survival and average VA gains, at the expense of systemic immunosuppression. Wang et al. (2019) reported KM success as 71.4% with allogeneic CLET (ACLET) versus 52.9% with COMET (*p* = 0.043); PED occurred more often after COMET, and eyelid disease increased failure risk [18]. In a three-arm series, Kengpunpanich et al. (2024) found SLET 77.8% (7-year survival 72.2%) outperforming COMET 57.8% (7-year 53.2%) and CLET 45.5% (7-year 50.0%). In this study SJS/TEN and Schirmer <5 mm predicted failure [2]. Elalfy et al. (2025) observed 81.7% stable surfaces with ACLET versus 60.7% with COMET at the last follow-up. ACLET eyes gained vision on average, whereas COMET eyes did not show a statistically significant mean VA change at the final visit in that dataset [17]. Τhese findings arise exclusively from non-randomised observational studies and must be interpreted with caution. Selection bias is likely, as COMET is often reserved for eyes with bilateral disease, severe ocular surface inflammation, or contraindications to systemic immunosuppression. In several cohorts, follow-up for COMET-treated eyes was substantially shorter than for limbal-derived procedures, reflecting real-world practice patterns and referral pathways. Given that LSCD recurrence may occur years after initial reconstruction, shorter observation windows may underestimate late failure rates and preclude definitive comparison of long-term durability. Comparative findings should therefore be interpreted as observational associations rather than evidence of procedural superiority.

### 3.10. Fornix Reconstruction in Cicatrising Disease

Where adnexal reconstruction was required, COMET-based fornix reconstruction produced durable anatomical success. Komai et al. (16 eyes) reported 5-year success 79.6% (KM). Absence of recurrence at 24 weeks predicted excellent 3-year survival (100% versus 33.3% if recurrent). These complex cases often required coordinated eyelid and surface procedures, while outcomes hinged on early inflammation control [10].

### 3.11. Safety

Serious adverse events were uncommon across COMET series. Overall, 65% of treated eyes had no reported complications. The most common complication was elevated intraocular pressure (93 eyes, 11%), largely attributable to prolonged steroid use. Corneal melting/perforation occurred in 60 eyes (7.0%), PEDs in 47 (5.5%), and infection in 42 (4.9%). Other reported events included atrophia 3 (0.4%), glaucoma 1 (0.1%), immunosuppression-related events 9 (1.1%), pain 9 (1.1%), and rejection 14 (1.6%). In Wang et al.’s study, rates of neovascularisation, corneal conjunctivalisation, and symblepharon improvement were similar between ACLET and COMET [18]. Reporting of neovascularisation varied considerably across studies and was inconsistently classified as a complication. While peripheral corneal neovascularisation was frequently observed, it was often transient or stabilised over time and not uniformly linked to graft failure. Nonetheless, given its clinical relevance for long-term transparency and visual rehabilitation, inconsistent reporting of neovascularisation represents an important limitation of the current literature. Donor-site oral morbidity was infrequently reported and generally limited to transient discomfort or minor mucosal symptoms. No study identified long-term oral complications, dysplasia, or malignancy; however, systematic surveillance and standardised reporting were limited.

### 3.12. Predictors of Outcome

Table 4 shows the predictors of outcome. Across cohorts, prognosis was worse with SJS/TEN or OCP, in eyes with a preoperative epithelial defect, severe tear deficiency (Schirmer < 5 mm), significant adnexal disease (entropion/trichiasis/symblepharon), or persistent early inflammation. Failures of biomaterial-free constructs clustered in eyes with four-quadrant symblepharon. Better outcomes were seen when the ocular surface was quiet at surgery, epithelialisation was rapid, adnexal disease was addressed, and—in some series—when substrate-free constructs were used.

## 4. Discussion

This scoping review maps two decades of clinical experience with cultivated oral mucosal epithelial transplantation (COMET/CAOMECS) for limbal stem cell deficiency (LSCD) across 893 eyes and 821 patients, largely from Asian and European centres, with aetiology dominated by chemical/thermal burns and SJS/TEN. Three important points are clear and directly relevant to practice. First, anatomical control is achievable but heterogeneous. At the study level, AM-based series typically reported mid-range 12-month success (~55–70%), when success is defined as a stable, epithelialised cornea without progressive conjunctivalisation or uncontrolled neovascularisation [8,9,23,32,34]. In contrast, when aggregated across COMET-treated eyes in this review, the proportion with a stable surface at the last follow-up was 45% (211/467). The discrepancy between mid-range 12-month success rates reported in individual series and the lower proportion of stable surfaces at the last follow-up reflects both late attrition over extended observation and heterogeneity in outcome definitions. Several studies reporting favourable 12-month outcomes demonstrated progressive decline in Kaplan–Meier survival curves beyond the first year, with failures accruing due to late recurrent epithelial breakdown, fibrovascular ingrowth, or persistent inflammation. Accordingly, the 45% figure should be interpreted cautiously (descriptive purposes).

Second, vision improves for most patients, with a mean improvement from 1.8 ± 0.7 to 1.4 ± 0.7 logMAR and ≥2-line gains in 65.4% (308/471). However, functional recovery remains constrained by pre-existing stromal opacity and vascularisation in cicatrising phenotypes, particularly in SJS/TEN and OCP. Third, technique matters. A matched comparison favoured substrate-free (fibrin-dish) COMET over AM-carried sheets for 12-month survival, neovascularisation, and BCVA [24]. A biomaterial-/carrier-free, feeder-free approach achieved reconstruction in most eyes while highlighting the risk posed by four-quadrant symblepharon [29]. These platform effects plausibly reflect differences in sheet integrity, trophic signalling, and the host–graft interface.

Against contemporary alternatives, the comparative cohorts provide a consistent message, that limbal-derived epithelium (ACLET/CLET/SLET) delivers higher surface survival and, on average, greater visual gain than COMET, but requires systemic immunosuppression [2,17,18]. In bilateral LSCD where immunosuppression is undesirable or unsafe, these data support COMET as an autologous, immunosuppression-sparing option with predictable safety. Notably, in complex cicatrising eyes requiring fornix reconstruction, COMET-based surgery produced durable anatomical success (5-year success of 79.6%), provided that early recurrence was prevented [10].

Early AM-supported sheets remain widely reported and practical [8,9,23,32,34]. Findings from Kyoto studies suggest substrate-free fibrin-dish constructs may temper early fibrovascular encroachment and improve early BCVA relative to AM [24]. Biomaterial-/feeder-free constructs are feasible, albeit with slower epithelialisation when the adnexa are severely cicatrised [29]. As regulators converge on xenobiotic-free manufacture and potency metrics, aligning on quality attributes—e.g., clonogenic content and p63α^bright proportion—should improve between-centre comparability and trial readiness [5,12].

Across series, outcomes were the best when surgery was performed on a quiet surface after meticulous control of eyelid disease and tear deficiency, and when early epithelial closure was achieved [9,23]. Early PED was the dominant cause for failure and a harbinger of longer-term breakdown [9,18]. In cicatrising disease, coordinated adnexal surgery (entropion correction, mucous-membrane grafting, symblepharon release) was integral to success [10,23]. Staged optical keratoplasty can be considered after stable epithelialisation with several series reporting favourable outcomes once the surface is quiescent, under careful anti-inflammatory strategies [23,31]. Limbal-vaulting rigid lenses can further improve function once inflammation is controlled [25].

Prolonged epithelialisation observed with biomaterial-free constructs raises important clinical considerations. Extended periods of epithelial vacancy may increase susceptibility to stromal inflammation, melt, infection, and secondary scarring, particularly in eyes with severe tear deficiency or adnexal disease. In the reported biomaterial-free cohorts, these risks were mitigated through intensive topical therapy and careful patient selection; nevertheless, slower epithelial closure likely contributes to early vulnerability and may partially explain clustering of failures in eyes with extensive symblepharon or active cicatrisation.

In acute PEDs, COMET achieved prompt closure in small but consistent cohorts, supporting its use as a rescue epithelial cover when conventional measures fail [14,33]. In fornix reconstruction for chronic cicatrising disease, stability was achievable and absence of recurrence by 24 weeks predicted excellent 3-year survival [10]. For aniridia-associated keratopathy, epithelial phenotype and VA gains were encouraging in a moderate-sized series, although long-range clarity remains uncertain in the face of progressive stromal change [22].

The safety profile across series was acceptable. Two-thirds of eyes had no reported complications. Steroid-related intraocular pressure rise was the most common event (11%), and rates of corneal melt/perforation (7.0%), PEDs (5.5%), and infection (4.9%) were low in absolute terms. Neovascularisation—often peripheral and self-limiting—was common but not consistently classified as a complication. No donor-site oncologic complication was detected. In one comparative cohort, rates of neovascularisation, corneal conjunctivalisation, and symblepharon improvement were similar between ACLET and COMET [18]. These findings align with COMET’s attraction in bilateral disease: autologous tissue, minimal systemic exposure, and a complication spectrum dominated by modifiable postoperative inflammation and steroid use.

Several predictors of failure recur across the literature and are practical for patient selection and counselling. SJS/TEN and OCP, a preoperative epithelial defect, Schirmer < 5 mm, significant adnexal disease, and early persistent inflammation all lead to poorer outcomes. Conversely, a quiet ocular surface at surgery, prompt epithelial closure, and, in some series, the use of substrate-free constructs were associated with success. In practice, and aligned with contemporary consensus pathways [3,4], COMET is best positioned for bilateral LSCD when long-term immunosuppression is undesirable or unsafe, whereas SLET/CLET/ACLET should be prioritised when donor tissue and immunosuppression are acceptable. Whatever the chosen modality, peri-operative optimisation with aggressive control of inflammation, tear film augmentation, and eyelid rehabilitation, likely exerts as much influence on success as the cell source itself.

This review has limitations inherent to the evidence base which includes small to moderate sample sizes, centre-specific case-mix, non-standardised postoperative regimens, and heterogeneous outcome definitions (fixed-time proportions vs. Kaplan–Meier survival). Short-term definitions of anatomical success have limited predictive value for long-term outcomes. Most series originated from Asia and Europe, which may limit generalisability to other settings. Visual acuity methods were inconsistently reported (e.g., low-vision conversions), limiting quantitative comparison across studies. We therefore avoided pooling across discrepant endpoints and instead we report study-level results and a conservative COMET-wide proportion at the last follow-up. Finally, follow-up for several cohorts was shorter in COMET than in comparator groups, which can bias between-arm inferences. We acknowledge that anatomical success was reported using fixed-time endpoints, Kaplan–Meier survival estimates, or last-visit outcomes, limiting comparability across cohorts. Thus, findings should be interpreted as descriptive and hypothesis-generating.

Cabral and colleagues [35] synthesised the pre-2020 COMET literature, highlighting variable definitions of success, heterogeneity in carriers and culture conditions, and the absence of head-to-head trials. Our scoping review extends that evidence map through 2025, incorporating later Kyoto/TDC updates, biomaterial-/feeder-free clinical experience, and three comparative cohorts juxtaposing COMET with limbal-derived procedures [2,17,18], which consistently show higher ocular surface survival and greater average visual gains with limbal epithelium at the cost of systemic immunosuppression. We also explicitly separate non-cultivated oral mucosal transplantation (OMET/SOMET) from COMET to avoid inappropriate pooling; the largest OMET cohort reports high anatomical stability with modest visual gains and should be analysed independently [13,20].

## 5. Conclusions

COMET is a mature autologous technology that offers immunosuppression-sparing ocular surface reconstruction for appropriately selected patients with bilateral LSCD. The weight of evidence supports moderate rates of durable anatomical stability, particularly when surgery is timed to a quiet surface and adnexal disease is addressed. Platform refinements (especially substrate-free constructs) and rigorous comparative studies are poised to clarify the boundaries of COMET’s advantage relative to limbal-derived epithelium and to optimise visual rehabilitation in this complex patient population.

## Figures and Tables

**Table 1:** Study characteristics: setting (country/centre), study design and sample size (eyes and patients) for each included report.

Study (Year)	Country/Setting	Design	Eyes (pts)
**Ang et al., 2006** [30]	Singapore (tertiary)	Prospective interventional case series	10 (10)
**Dobrowolski et al., 2015** [22]	Poland (single-centre)	Retrospective case series	17 (13)
**Aziza et al., 2024** [25]	Japan (multicentre)	Retrospective, adjunct therapy after COMET (limbal-rigid CL)	23 (18)
**Booranapong et al., 2022** [26]	Thailand	Small clinical series (methods + 6 pts longitudinal)	6 (6)
**Burillon et al., 2012** [32]	France (multicentre)	Prospective, Gehan’s two-stage	26 (25)
**Gopakumar et al., 2019** [33]	Japan	Mixed cohorts: acute-stage LSCD (n = 6) and fornix reconstruction in cicatrizing disease (n ≈ 19)	25 (24)
**Elalfy et al., 2025** [17]	UK tertiary corneoplastic unit	Comparative real-world cohort: COMET vs. allogeneic CLET (ACLET)	COMET 41 vs. ACLET 69
**Hirayama et al., 2012** [24]	Japan (Kyoto)	Case–control comparison: substrate-free (fibrin) vs. AM-based COMET	32 (32)
**Inatomi et al., 2006** [34]	Japan (Kyoto)	Prospective case series	15 (12)
**Kengpunpanich et al., 2024** [2]	Thailand (Siriraj)	Retrospective comparative (CLET vs. SLET vs. COMET)	103 eyes (94 pts) overall
**Kim et al., 2018** [29]	Korea (SNUH)	Prospective controlled clinical trial	8
**Komai et al., 2022** [10]	Japan (Kyoto)	Retrospective cohort (fornix reconstruction)	16 (15)
**Ma et al., 2009** [31]	Taiwan	Prospective feasibility series	5 (5)
**Nakamura et al., 2004** [8]	Japan (Kyoto)	Early prospective case series	6
**Nakamura et al., 2011** [27]	Japan (Kyoto)	Long-term cohort	19 (17)
**Nakamura et al., 2007** [19]	Japan (Kyoto)	Mechanistic (removed graft histology)	6 eyes (5 pts)
**Prabhasawat et al., 2016** [23]	Thailand (Siriraj)	Prospective non-comparative series	20 (18)
**Priya et al., 2011** [15]	India (Aravind)	Translational + small clinical	10 (10)
**Satake et al., 2011** [9]	Japan (Tokyo Dental College)	Retrospective interventional	40 (36)
**Shimazaki et al., 2020** [16]	Japan (multicentre)	Retrospective cohort (CCST)	162 eyes (246 surgeries) total CCST; COMET n = 143 within cohort (analysed per eye)
**Sotozono et al., 2014** [14]	Japan (Kyoto)	Retrospective series (PED in severe OSD)	10 (9)
**Sotozono et al., 2013** [28]	Japan (Kyoto)	Retrospective outcomes	46 (40)
**Venugopal et al., 2021** [21]	India (Tertiary SJS service)	Prospective interventional	45 (41)
Zhu et al., 2023 ** [20]	China (Sir Run Run Shaw Hospital, Zhejiang Univ.)	Retrospective series	49 eyes (48 pts)
**Wang et al., 2019** [18]	China (PLA General Hospital; Shandong Eye Institute)	Retrospective comparative cohort (ACLET vs. COMET)	76 eyes (73 pts)—ACLET 42 eyes (41 pts); COMET 34 eyes (32 pts)

COMET, cultivated oral mucosal epithelial transplantation; CLET, cultivated limbal epithelial transplantation; ACLET, allogeneic CLET; SLET, simple limbal epithelial transplantation; CCST, cultivated cell sheet transplantation programme; AM, amniotic membrane; pts, patients. ** Zhu et al.’s study on OMET (non-cultivated oral mucosa) was included for context, and not pooled with COMET outcomes.

**Table 2:** Aetiology of limbal stem cell deficiency by study.

Study (Year)	LSCD Aetiology
**Ang et al., 2006** [30]	SJS/TEN × 7, thermal injury × 1, chemical injury × 1, OCP × 1
**Dobrowolski et al., 2015** [22]	17 congenital aniridia with total/subtotal LSCD
**Aziza et al., 2024** [25]	SJS/TEN × 21, OCP × 2
**Booranapong et al., 2022** [26]	SJS/TEN × 3, chemical burns × 3
**Burillon et al., 2012** [32]	Corneal burn × 9, neuroparalytic keratitis × 2, rosacea keratitis × 3, Lyell syndrome × 4, severe trachoma × 1, contact lens hypoxia × 1, congenital aniridia × 1, cystinosis × 1, severe Groenouw dystrophy × 1, hepatitis C × 1, contact lens hypoxia + congenital aniridia × 2
**Sotozono et al., 2014** [14]	SJS/TEN × 3, thermal injury × 3, chemical injury × 2, OCP × 2
**Gopakumar et al., 2019** [33]	SJS/TEN × 11, chemical injury × 12, OCP × 1
**Elalfy et al., 2025** [17]	Aniridia × 47, SJS × 9, thermal injury × 4, chemical injury × 15, OCP × 1, rosacea × 9, other × 12
**Hirayama et al., 2012** [24]	Chemical 6, pseudo-OCP 6, SJS 2, OCP 2 per group
**Inatomi et al., 2006** [34]	SJS/TEN × 7, chemical injury × 5, thermal injury × 1, pseudo-OCP × 1, idiopathic × 1
**Kengpunpanich et al., 2024** [2]	Unspecified burn × 44, SJS/TEN × 27, other × 32, allergic conjunctivitis × 5, PUK × 7, multiple surgeries × 4, MGD × 3, MMC toxicity × 4, aniridia × 3, pterygium × 2, ocular trauma × 1, idiopathic × 1
**Kim et al., 2018** [29]	SJS/TEN × 6, chemical injury × 1, OCP × 1
**Komai et al., 2022** [10]	OCP × 5, thermal × 2, chemical × 1, SJS × 1, pterygium × 2, GVHD × 1, Trachoma × 1, POCP × 1, Idiopathic × 1
**Ma et al., 2009** [31]	Chemical burn × 3, thermal burn × 2
**Nakamura et al., 2004** [8]	SJS/TEN × 3, chemical burns × 3
**Nakamura et al., 2011** [27]	SJS/TEN × 11, chemical or thermal injury × 1, OCP × 4, squamous cell carcinoma × 2, GVHD × 1
**Nakamura et al., 2007** [19]	SJS/TEN × 3, chemical injury × 3
**Prabhasawat et al., 2016** [23]	SJS/TEN × 10, chemical burn × 7, multiple eye surgery × 1, advanced pterygium × 1, ocular trauma × 1
**Priya et al., 2011** [15]	SJS/TEN × 1, chemical injury × 9
**Satake et al., 2011** [9]	SJS/TEN × 12, chemical or thermal injury × 11, OCP × 9, pOCP × 7, gelatinous drop-like dystrophy × 1
**Shimazaki et al., 2020** [16]	SJS/TEN × 41; burns 52; OCP × 32; others × 18
**Sotozono et al., 2014** [14]	SJS/TEN × 3, thermal injury × 3, chemical injury × 2, OCP × 2
**Sotozono et al., 2013** [28]	SJS/TEN × 21, OCP × 10, chemical or thermal injury × 7, idiopathic × 3, radiation keratopathy × 1, GVHD × 1, congenital aniridia × 1, Salzmann’s corneal degeneration × 1, drug toxicity × 1
**Venugopal et al., 2021** [21]	Chronic SJS/TEN sequelae × 45
Zhu et al., 2023 ** [20]	Chemical 30, thermal 16, explosive 1, SJS/TEN 1, multiple pterygia 1
**Wang et al., 2019** [18]	Chemical injury × 16, thermal injury × 18

LSCD, limbal stem cell deficiency; SJS/TEN, Stevens–Johnson syndrome/toxic epidermal necrolysis; OCP, ocular cicatricial pemphigoid; pOCP, pseudo-OCP; GVHD, graft-versus-host disease; PUK, peripheral ulcerative keratitis; MGD, meibomian gland dysfunction. ** Zhu et al.’s study on OMET (non-cultivated oral mucosa) was included for context, and not pooled with COMET outcomes.

**Table 3:** Comparative cohorts (effect sizes and survival measures). Head-to-head comparative cohorts including a COMET arm: study arms (eyes), follow-up, the success metric used by each study, and the principal effect estimate or group comparison.

Study	Arms (Eyes)	Follow-Up	Success Measure	Key Result
Wang 2019 [18]	ACLET 42 vs. COMET 34	ACLET 23.3 9.9 mo; COMET 16.1 ± 5.8 mo	KM stable ocular surface	71.4% ACLET vs. 52.9% COMET (*p* = 0.043); PED more frequent after COMET; eyelid disease increased failure
Kengpunpanich 2024 [2]	CLET vs. SLET vs. COMET (103 total)	Median 75 mo	Surgical success + KM	SLET 77.8% (7-y 72.2%); COMET 57.8% (7-y 53.2%); CLET 45.5% (7-y 50.0%); SJS/TEN & Schirmer <5 mm → failure
Elalfy 2025 [17]	ACLET 69 vs. COMET 41	ACLET 18 mo; COMET 5 mo	Stable surface at last visit	81.7% ACLET vs. 60.7% COMET; ACLET had significant VA gain

ACLET, allogeneic cultivated limbal epithelial transplantation; CLET, cultivated limbal epithelial transplantation; SLET, simple limbal epithelial transplantation; COMET, cultivated oral mucosal epithelial transplantation; KM, Kaplan–Meier (time-to-event); VA, visual acuity; mo, months; y, years; pts, patients.

**Table 4:** Predictors of outcome reported across studies. Study-specific predictors associated with success or failure of ocular surface reconstruction after COMET/CAOMECS, with the analytic frame used in each report and the direction of the observed signal.

Study	Cohort Context	Predictor	Analysis	Key Findings
Satake 2011 [9]	Total LSCD AM-based	Pre-op epithelial defect--> early failure	Observed association	Early window is critical
Hirayama 2012 [24]	Substrate-free vs. AM	Substrate-free: increased survival + better BCVA	KM; *p* = 0.046	Technique effect
Wang 2019 [18]	ACLET vs. COMET	Eyelid abnormalities: increased failure’ PED more frequent after COMET	KM; group comparison	PED dominant failure mode
Kengpunpanich 2024 [2]	3 arm cohort	SJS/TEN, Schirmer < 5 mm: failure	Risk model	Cicatrizing dry eye high risk
Komai 2022 [10]	Fornix reconstruction	Recurrence by 24 week: poor long-term survival	KM stratified	Early control predicts outcomes
Kim 2018 [29]	Biomaterial-free	4 quadrant symblepharon: failure	Series observation	Adnexal burden matters
Shimazaki 2020 [16]	CCST programme	Pre-op Epi defect: failure	Programme analysis	Concordant with the COMET literature

KM, Kaplan–Meier; PED, persistent epithelial defect; BCVA, best-corrected visual acuity; SJS/TEN, Stevens–Johnson syndrome/toxic epidermal necrolysis.

## Data Availability

Data is available from the corresponding author upon reasonable request.
